# Supplementary material for: SIMPLICITY is an agent-based, multi-scale mathematical model to study SARS-CoV-2 intra- and between-host evolution
Source: Commun Biol. 2026 Jan 9;9:124. doi: 10.1038/s42003-025-09403-y (PMC12855898; doi:10.1038/s42003-025-09403-y)
Supplement: Supplementary file 2 — Supplementary Tables and Figures [file 42003_2025_9403_MOESM2_ESM.pdf]

Supporting Information

Table S1. Simulation Parameters for OSR fit. The nucleotide substitution rate (NSR) was the variable parameter in this set of simulations, taking 15 values logarithmically spaced from 1×10^{-6} to 3×10^{-4}, $\tau_1, \ldots, \tau_4$ denote the expected residence times from the inter-host model in days.

Parameter	Value
Population Size	1000
Initial Infected Individuals	10
Final Time	1095
Basic Reproduction Number (R)	1.1
τ_1	2.86
τ_2	3.91
τ_3	7.5
τ_4	8
Diagnosis Rate	0.1
Intra Host Virus Emergence Rate	0
Phenotype Model	immune waning
Sequencing Rate	0.05
Max Runtime (seconds)	86000

Table S2. Comparison of scores for the different OSR fitting curves. Akaike information criterion (AIC), Baysian information criterion (BIC) and coefficient of determination (R^2). Exp, log, spline, lin, tan relate to exponential, logarithmic, splines, linear and tangent function, respectively, relating nucleotide substitution rate to observed substitution rate (OSR).

Model	AIC	BIC	R^2
exp	−12 437.29	−12 423.53	0.82
log	−12 423.19	−12 409.43	0.81
spline	−12 249.25	−12 221.74	0.76
lin	−11 946.13	−11 936.96	0.64
tan	−11 942.13	−11 923.79	0.64

Table S3. Definitions of variables in the SIMPLICITY core algorithm (Extrande)

Variable	Type	Description
t	Variable	The current time of the simulation.
t_0	Constant	The initial start time of the simulation.
t_{final}	Constant	The end time for the simulation.
Δt_{acc}	Variable	An accumulator for time steps. It triggers periodic updates (e.g., mutations) when it exceeds a threshold.
L	Variable	The look-ahead time. Represents the maximum safe time step before reaction rate bounds must be recalculated.
B	Variable	The computed upper bound for the sum of all reaction propensities (rates) in the system at time t.
Δt	Variable	Time step to next event drawn from an exponential distribution.
δ_{min}	Constant	The minimum time interval required before periodic processes like mutation are executed.
reaction_id	Variable	An identifier for the reaction happening during an accepted step.
event_type	Variable	A descriptor for the type of event that occurred in a step (e.g., 'leap' or 'reaction').
population	Object	A data structure holding the state of all individuals in the simulation.
reporter	Object	An object responsible for logging, monitoring, and saving simulation output.

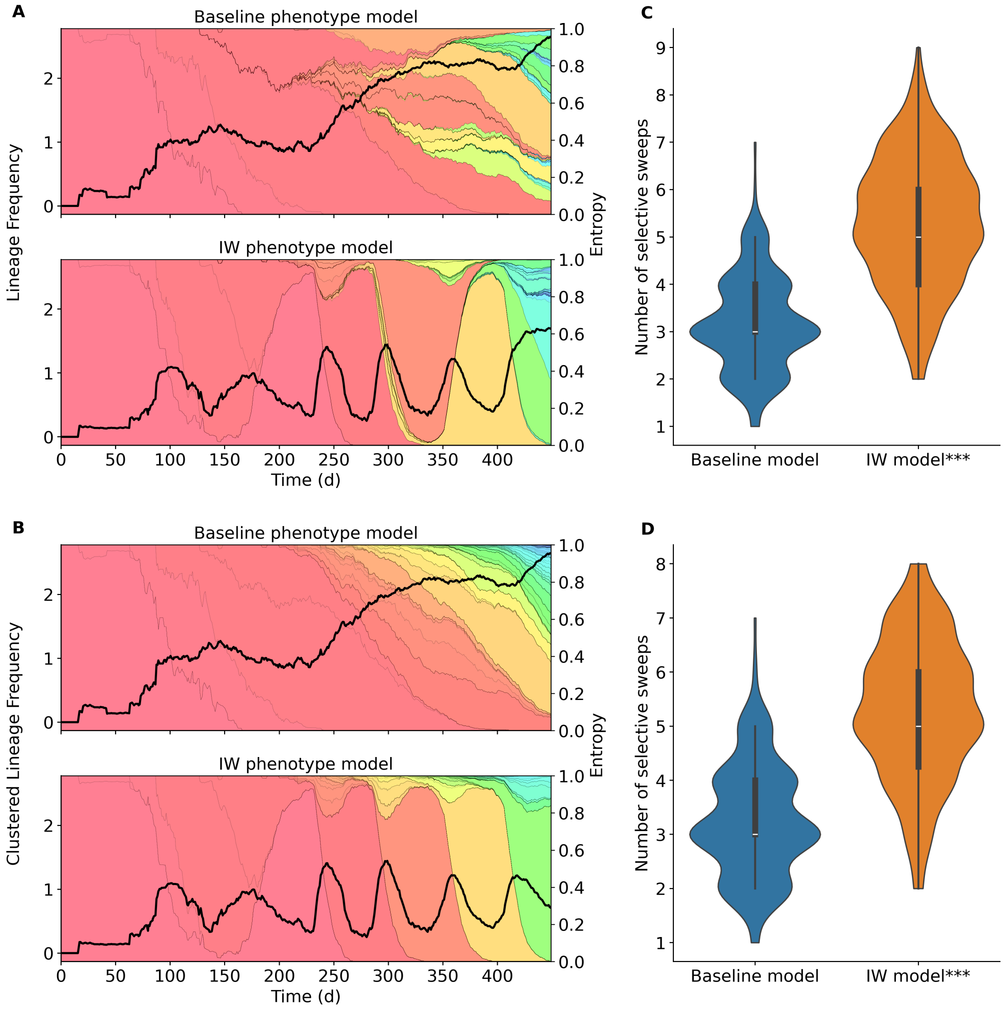

Fig S1. Extended violin plot of system entropy and selective sweeps. A, B Entropy trajectory from a single simulation (random seed = 7) under the baseline model and the immune-waning model. Lineages in **B** are clustered if they share at least 5 substitutions. **C, D** Violin plots comparing the distribution of selective sweep counts between the two models across multiple simulations (N=206). Boxplots encompass the inter-quartile ranges (IQR), central dots depict the median and whiskers extend to 1.5 times the IQR. Lineages in **D** are clustered if they share at least 5 substitutions.

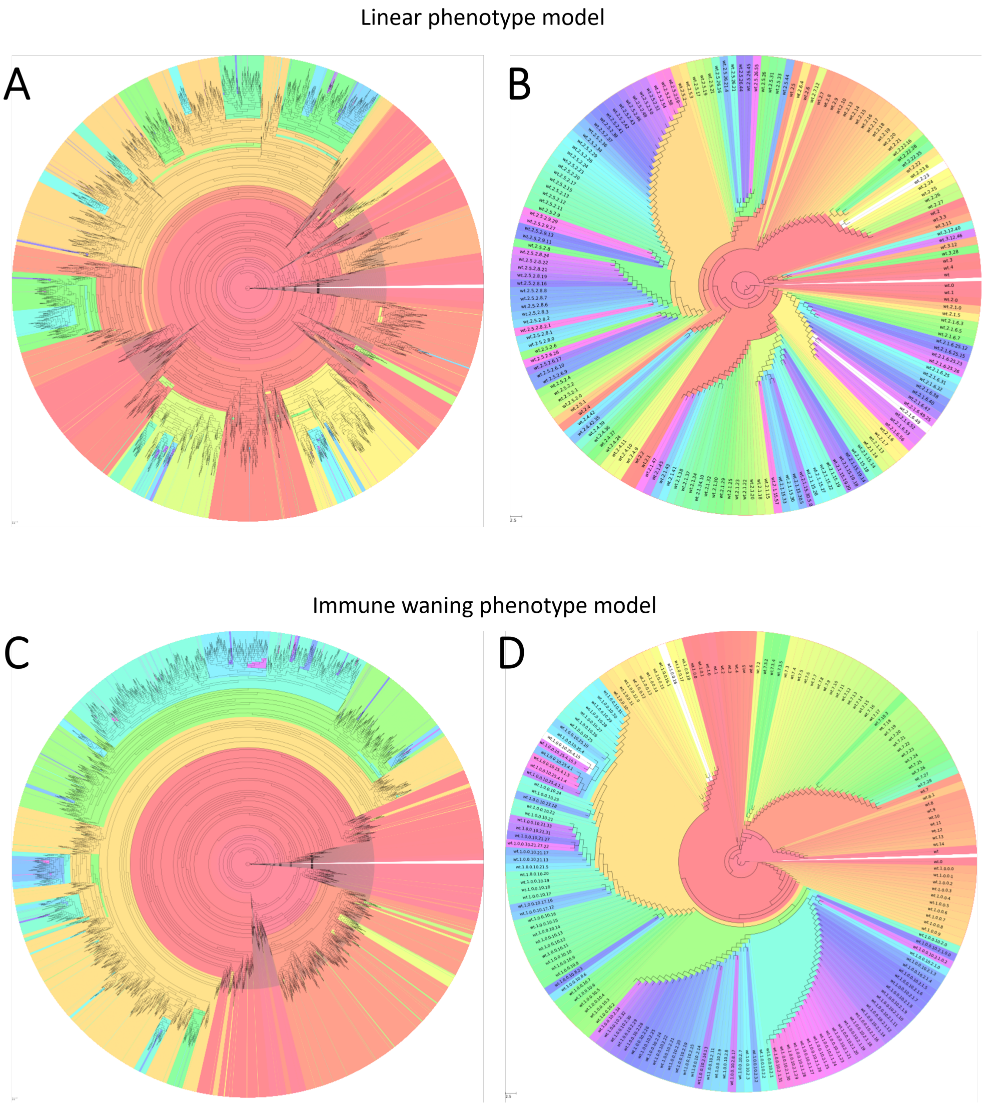

Fig S2. Infection and phylogenetic trees for linear and immune-waning phenotype models in seeded simulations. A Infection tree for the linear phenotype model. **B** Phylogenetic tree for the linear phenotype model. **C** Infection tree for the immune-waning phenotype model. **D** Phylogenetic tree for the immune-waning phenotype model.

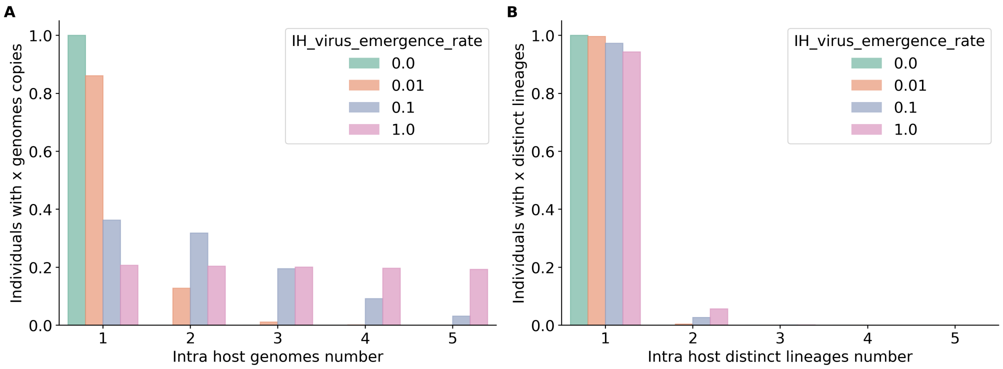

Fig S3. Intra-host lineage diversity for different values of virus emergence rates. A Density of the number of genomes per host across different virus emergence rates (k_v). **B** Density of the number of distinct lineages per host across different virus emergence rates (k_v).
